# Projecting groundwater storage changes in California’s Central Valley

**DOI:** 10.1038/s41598-018-31210-1

**Published:** 2018-08-27

**Authors:** Elias C. Massoud, Adam J. Purdy, Michelle E. Miro, James S. Famiglietti

**Affiliations:** 1 Department of Civil & Environmental Engineering, University of California, Irvine, CA 92697 USA; 2 Jet Propulsion Laboratory, California Institute of Technology, Pasadena, CA 91109 USA; 3 Department of Earth System Sciences, University of California, Irvine, CA 92697 USA; 4 Department of Civil & Environmental Engineering, UCLA, Los Angeles, CA 90024 USA

## Abstract

Accurate and detailed knowledge of California’s groundwater is of paramount importance for statewide water resources planning and management, and to sustain a multi-billion-dollar agriculture industry during prolonged droughts. In this study, we use water supply and demand information from California’s Department of Water Resources to develop an aggregate groundwater storage model for California’s Central Valley. The model is evaluated against 34 years of historic estimates of changes in groundwater storage derived from the United States Geological Survey’s Central Valley Hydrologic Model (USGS CVHM) and NASA’s Gravity Recovery and Climate Experiment (NASA GRACE) satellites. The calibrated model is then applied to predict future changes in groundwater storage for the years 2015–2050 under various precipitation scenarios from downscaled climate projections. We also discuss and project potential management strategies across different annual supply and demand variables and how they affect changes in groundwater storage. All simulations support the need for collective statewide management intervention to prevent continued depletion of groundwater availability.

## Introduction

California has a complex and storied history of water management. A statewide plan of water storage, infrastructure, and conveyance has ensured for many decades a steady supply of surface water to satisfy demands of the more arid Central and Southern parts of the state. However, in recent years, urban, agricultural, and environmental demands in California have exceeded the natural renewable supply. To date, this gap between the available statewide surface water supply and the growing water demand has been met primarily by extraction of non-renewable groundwater resources, which includes all forms of groundwater stored below the vadose zone and encompasses both phreatic and confined water. Yet, this pragmatic solution can have dire consequences as continued groundwater extraction depletes subsurface reservoirs, an environmental consequence that is being observed globally in semi-arid regions with highly-variable precipitation^1–7^. The protection of California’s groundwater resources is critical for sustaining the state’s livelihood, ecology, and agricultural production, and is key to preventing potentially harmful regional economic impacts that severe water shortages can cause.

**Figure 1:**
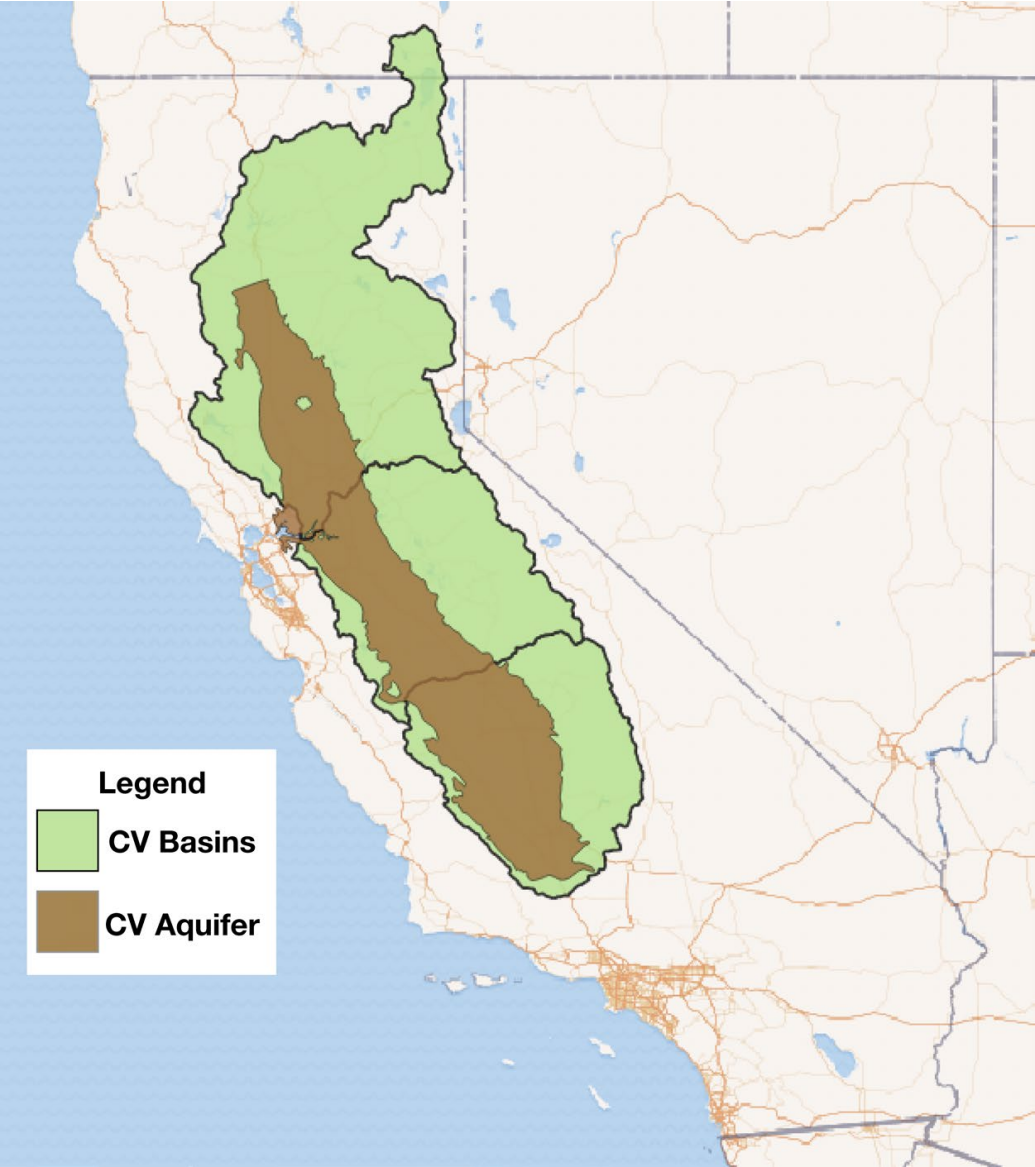
The Sacramento (upper), San Joaquin (middle), and Tulare (bottom) basins and respective aquifers, together forming the Central Valley in California. The basin boundary in green indicates the areas used to downscale the precipitation projections. The aquifer boundary in brown indicates the regions where the aggregated groundwater storage changes are represented in this study.

California’s Central Valley (CV), depicted in Fig. 1, is the most productive agricultural area in the United States and has already witnessed significant groundwater depletion. Annually, at least 40 percent or more of the CV’s water supply comes from groundwater, which is primarily used to meet agricultural demand^8^. The groundwater extracted for irrigation often exceeds the natural recharge, leading to declines in the groundwater table^9–12^. This impact has been even more pronounced during prolonged dry periods when groundwater reliance increases. In addition to agriculture, the CV has a growing population that increases demands on the region’s water resources. Data from the United States Geological Survey (USGS) shows that between 1962 and 2003 groundwater use increased from 0.75 to 2.5 km^3^ per year^9^. This mounting reliance on groundwater has continued with depletion rates as high as 32 mm per year despite the implementation of various urban conservation measures and an increasing use of surface water^4,9,10^.

Although livelihoods in and the economy of the CV depend in large part on the availability of groundwater, detailed year-to-year data on how much water is being extracted and used is largely lacking, particularly when viewed in comparison to surface water resources. Groundwater monitoring networks do not exist at the same scope and scale for those that track surface water^13^. In fact, no comprehensive framework for monitoring the world’s groundwater resources currently exists^5^. To bypass the lack of publicly available well monitoring data, various methods have been used and developed to estimate groundwater storage. This includes geostatistical interpolation methods and computer model simulations^14^. Furthermore, in recent years, much effort has focused on groundwater monitoring using remote sensing data^1,2,4,12,15,16^. Yet, groundwater storage predicted by these various methods is subject to large uncertainties. Therefore, there is an urgent need for new monitoring techniques and computational methods to estimate groundwater resources at the temporal and spatial scale relevant to water management^17^.

In this study, we develop an alternative simplified method to estimate changes in groundwater storage in the CV using a historic record of annual water use and supply from California’s Department of Water Resources (DWR). Our Groundwater Depletion (GWD) model builds on DWR’s water balance and uses empirical relationships between annual precipitation, supply, and demand to simulate (predict) annual groundwater pumping as well as recharge rates at the aggregated scale of the CV. The simulated changes in groundwater storage of the GWD model is evaluated against historic data of changes in groundwater storage (1981–2014) derived from the USGS Central Valley Hydrologic Model (USGS CVHM) and NASA’s Gravity Recovery And Climate Experiment (NASA GRACE) satellites. The GWD model is then used to predict changes in groundwater storage for the years 2015–2050. During this period, we evaluate the impact of different precipitation scenarios on future simulated groundwater availability, and we also discuss potential management strategies and how they affect changes in groundwater storage.

The GWD model is a tool that incorporates various sources of data and associated uncertainties to estimate groundwater availability. Our model does not offer the process-based flexibility (i.e. by containing hundreds or thousands of parameters that represent different system properties) or detailed spatial and temporal information that the USGS CVHM provides, and it cannot deliver larger scale complementary observations like the NASA GRACE satellites can. Instead, it is a parsimonious means of analyzing how variability in precipitation interacts with multi-year water management decisions that ultimately determine groundwater storage fluctuations over time. Overall, the goals of this study are: (i) to develop a simple model that accurately captures historic groundwater storage changes in the complex CV basin; (ii) to project future changes in groundwater storage based on downscaled climate projections as well as different management strategies; and, (iii) to showcase the additive value of combining multiple data sources for generating insights on complex hydrologic systems.

## Results

**Figure 2:**
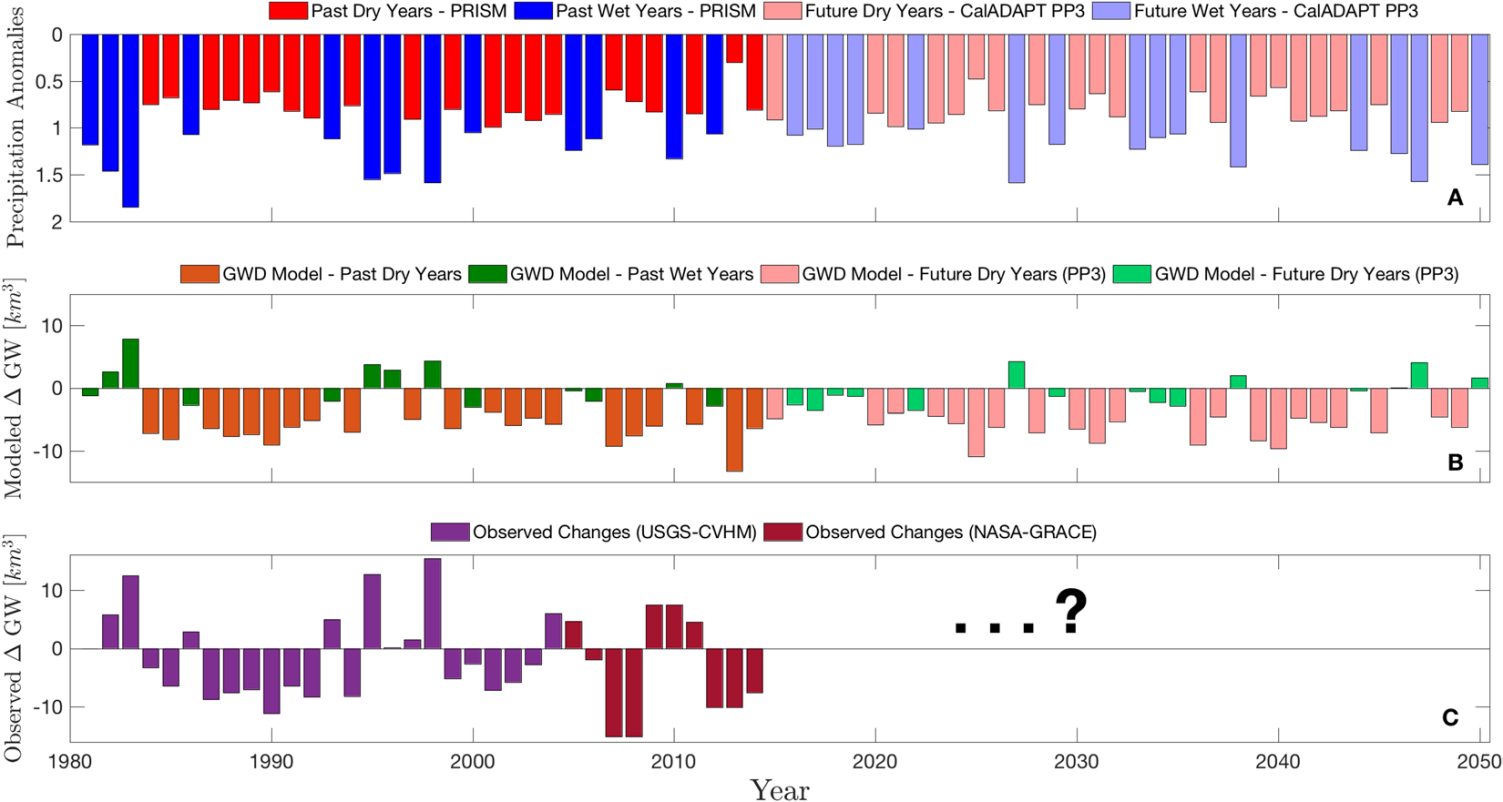
Yearly precipitation anomalies are shown in Panel A. PRISM data was used for the years 1981–2014 (dark colors) and Cal-Adapt data was used for the years 2015–2050 (light colors); dry years are shown in red and wet years are shown in blue. In Panel B, the change in simulated yearly groundwater storage is shown, which is calculated as the difference between the volume of water extracted from groundwater and the recharge that replenishes it each year. Panel C highlights the observed changes in groundwater levels in order to facilitate a direct comparison with the model’s estimated values in Panel B.

The model uses precipitation as the principal driver of groundwater storage changes. For estimating past conditions, precipitation data from the Parameter-Elevation Regressions on Independent Slopes Model (PRISM)^18^ is used to drive the model. For future simulations from 2015–2050, we employ four different precipitation scenarios obtained from the Cal-Adapt database to get a sense of uncertainty in our projections. The four models include the Community Earth System Model (CCSM3.0)^19^, the Centre National de Recherches Météorologiques sponsored CNRM-CM5^20^, the Geophysical Fluid Dynamics Laboratory (GFDL) model by the National Oceanic and Atmospheric Administration (NOAA)^21^, and the Parallel Climate Model (PCM) sponsored by the United States Department of Energy (USDOE)^22^. All data were aggregated to reflect yearly precipitation anomalies over the basins surrounding California’s CV, and are shown color coded in Fig. 2A to distinguish between wet and dry years.

### Estimating changes in groundwater storage of past years

The plot in Fig. 2B shows the change in simulated yearly groundwater storage, calculated as the difference between groundwater extraction and recharge to groundwater, respectively. Figure 2C shows the observed changes in groundwater storage from USGS and GRACE data sources and allows for a comparison with model results. Evident in Fig. 2B,C are large decreases in groundwater storage during dry years, which are likely the result of increased groundwater reliance and decreased natural recharge. The simulated changes in groundwater match the observed changes over periods of multi-year consecutive negative precipitation anomalies (e.g. drought in 1987–1992). The model simulations also show skill representing increases in groundwater storage (1981–1982 and 1995–1998), but these simulated increases are of lower magnitude than the observed changes.

**Figure 3:**
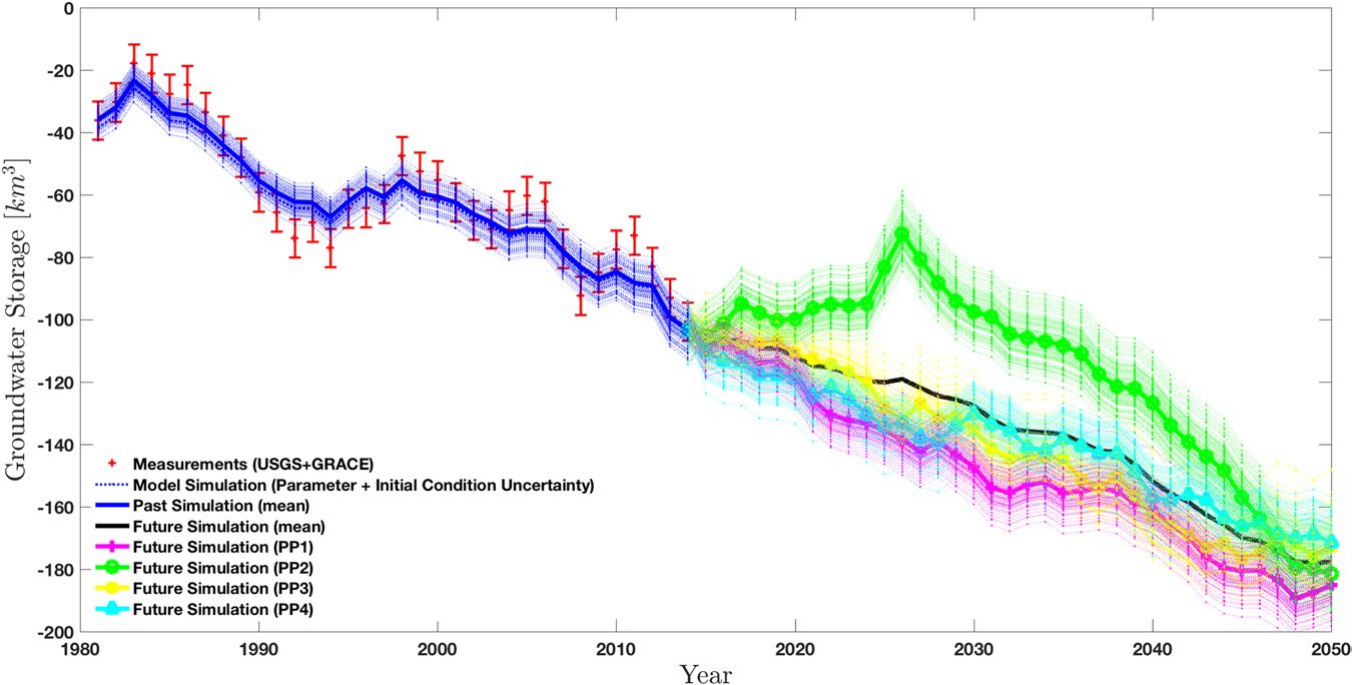
The GWD Model simulations for both the past and future years. The simulated groundwater storage changes for the years 1980–2014 (Blue line) are compared with observations (red icons). In future years, several precipitation scenarios are examined.

The model’s simulated changes in groundwater storage from 1980–2014 and its associated parametric and initial condition uncertainty are shown in Fig. 3 (blue lines). Our simulations are also compared with observations (red bars) for the years 1980–2014. Despite underestimating increases in groundwater during wet years, the mean model simulation matches the historical measured dataset (1981–2014) with a Root Mean Square Error (RMSE) of 6.8 km^3^ and a correlation coefficient of 0.9532. Given that a measurement error of 5 km^3^ was implemented, we consider this average error for the model simulation to be reasonable. Considering the model is structured to aggregate processes that occur at annual time-steps and over the spatial scale of the CV, the ranges of the groundwater storage change simulations fit within the uncertainty ranges of the observations for most years.

It is important to note that a possible reason for discrepancy between the GWD modeled change in groundwater storage and the GRACE-based observations could be from GRACE uncertainty^2,16^. GRACE-based groundwater storage change estimates rely on models to account for the non-groundwater components (i.e. soil moisture, surface water, and snowpack) of GRACE-derived changes in total water storage. These models often do not fully account for anthropogenic impacts on water storage changes in addition to having their own degree of uncertainty. These sources of error are propagated into GRACE groundwater storage changes^4^.

### Future projections of groundwater storage change

The projected change in groundwater storage during future years (2015–2050), shown in Fig. 3, is examined for various precipitation scenarios. Future precipitation projections were derived using data from Cal-Adapt. From these simulations, it is clear that if no changes are made in water management, then groundwater depletion will continue. This depletion is shown for all of the precipitation scenarios considered and is primarily due to an unbalanced water management portfolio. All future projections are based on current ‘business-as-usual’ management strategies. Based on the fit of historical data, we find the statewide strategies employed from 1998–2010 is also representative of water management since the 1980’s, and regional and local water management has been ineffective in stabilizing a regional water balance therefore causing depletion of the groundwater aquifer.

**Figure 4:**
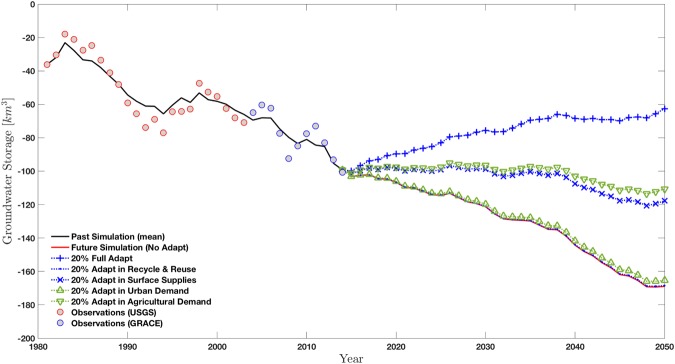
The mean changes in groundwater storage simulated for both past and future years. Several management scenarios are examined for future years. The case of no changes in local or regional water management is shown with a red line. Each supply/demand variable’s sensitivity to groundwater depletion is examined by assessing 20% changes in each variable individually, indicated by blue for supply changes and by green for demand changes. Finally, a combined strategy that incorporates 20% changes in all variables simultaneously is implemented (i.e. 20% augmentation in Recycle and Reuse and in Surface Supplies as well as 20% reduction in Urban and Agricultural Demands), and shown with blue crosses.

Since it is apparent that if no changes are made in water management then the groundwater depletion will continue, we examine several management scenarios for future years (2015–2050). We consider simple management options to depict how a change in annual supply and demand variables affect changes in groundwater storage. To get an idea of how sensitive groundwater storage is to each variable individually, we run the model with individual changes of 20% in each demand or supply variable, which translates to a 20% increase in supplies or decrease in demands, respectively, which would ultimately reduce the reliance on groundwater. In Fig. 4, the mean simulation of each of these scenarios is shown, along with a case of no changes in management (i.e. business-as-usual simulation) and the case with significant management intervention (i.e. 20% change in all considered variables). Model results show that increases in surface water supply and agriculture efficiency have a stronger potential to stabilize groundwater storage in the Central Valley basin compared to urban water use efficiency and increased supply from recycle and reuse. The ‘Full Adapt’ strategy results in the only scenario that allows for recovery of the whole groundwater system, while all other strategies show that depletion of the aquifer will continue into the coming decades. From these results it is clear that a comprehensive approach that looks at both supply and demand side management strategies, especially for surface supplies and agricultural demands, may be necessary to sustain groundwater levels in the future.

**Figure 5:**
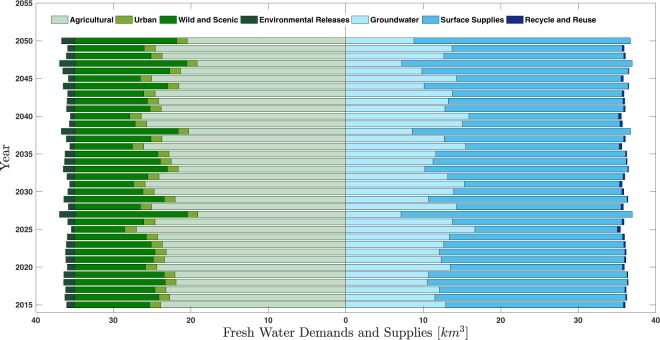
Butterfly chart obtained by driving the model with the PP3 forcing data. Displayed are projected estimates of the fresh water demands during each year and the respective fresh water supplies of each year. It is clear that during projected dry years (e.g. 2040), groundwater pumping is relatively high and during projected wet years (e.g. 2050) the pumping is noticeably lower.

Figure 5 provides a more in-depth look at the model simulations into the future. Shown in the figure is a butterfly chart obtained by driving the model with the PP3 forcing data. Figure 5 displays projected estimates of the fresh water demands during each year and the respective fresh water supplies of each year. It is clear that during projected dry years (e.g. 2040), groundwater pumping is relatively high and during projected wet years (e.g. 2050) the pumping is noticeably lower. This heavy reliance on groundwater during dry years is exactly the anthropogenic process that the GWD model is intended to capture.

## Discussion

Due to the high degree of uncertainty associated with inter-annual precipitation and surface water availability in semi-arid regions, groundwater is often relied upon to meet water needs. Because of this, consistent multi-year strategies are needed to better understand and effectively manage groundwater storage. Lack of current effective management is already apparent in regions where wells have run dry, subsidence is impacting infrastructure^23^, groundwater quality is degrading^24^, salinization of soil and groundwater resources is taking place^25,26^, and environmental impacts are becoming more visible^27^. Allowing unsustainable use of groundwater resources will only exacerbate these problems. Costs associated with water extraction will also increase due to the need to drill deeper wells, treat degraded groundwater quality and pump from deeper depths^28^. Moreover, 21 groundwater basins within California are classified as critically over-drafted, and recent groundwater management regulations require implementation of groundwater sustainability plans.

The GWD model is a useful tool to assess the water balance in the CV and provides useful information at aggregate spatial and temporal scales. Building from the current structure to model at the sub-basin, the scale at which groundwater management is now needed in California, can further the potential impact of this tool to help identify management scenarios that incorporate processes that occur at finer spatiotemporal scales not captured for the aggregate CV GWD model. For example, recent studies^12,13^ have highlighted spatial differences of groundwater depletion for regions within the CV, which can occur due to factors such as different precipitation rates or different spatial hydrogeologic parameters. Additionally, incorporating sub-basin observations of reservoir storage, a variable that can greatly differ across sub-basins, into the model structure can provide a more detailed picture on the multi-year dynamics of surface water management. Yet, for investigation of the spatial differences of groundwater depletion in the CV, a more complex and higher resolution analysis is required. Generally, the flexibility of our model provides California’s newly-formed Groundwater Sustainability Agencies a tool that inexpensively projects the impacts of changing precipitation regimes and management actions on changes in groundwater storage^29^.

The relatively constant gap between renewable surface water supplies and statewide demands indicates that strategies for increasing freshwater supplies and demand reduction strategies implemented today could have lasting impacts into the future. From our simulations it is clear that if no changes are made in water management, groundwater depletion in the CV will continue due to projected decreases in precipitation and an unbalanced water management portfolio. Agricultural efficiency, which encompasses more than just on-farm water use efficiency and includes other forms of demand management, seems to be a promising area to focus on to reduce heavy reliance on groundwater. Reductions in irrigation demand can also reflect changes in total farmed acreage. Also, the development of recycled and reused water systems and the improvement of urban water efficiencies could help close the gap even more and further reduce the reliance on non-renewable groundwater resources, but these actions will not support the state-wide agricultural demands. Overall, we believe that with a more comprehensive CV-wide supply and demand management strategy, groundwater storage can be properly managed, showing potential that this resource can indeed be sustained and secured for future generations to buffer the impacts from future droughts.

This study showcases the additive value of combining multiple data sources for predicting long-term dynamics in a natural system^30,31^. In comparison, other common methods for estimating groundwater storage changes are relatively costly, such as the USGS CVHM model or the NASA GRACE satellites. The GWD model developed in this study fused information from various sources, such as DWR, USGS, and NASA, and then used various other sources of information, such as precipitation estimates, to project aggregated system behavior. Furthermore, these projections were accompanied with formal estimates of uncertainty. The uncertainty sources that are explicitly considered are the model structural error that arises from the DWR supply and demand variables, the measurement error applied for the USGS and GRACE data, and the parametric uncertainty that was obtained for the groundwater recharge parameter through the Markov Chain Monte Carlo (MCMC) sampling. In all, this method achieves a parsimonious way to determine groundwater storage fluctuations over time with formal confidence bounds.

## Methods: The Empirical Groundwater Depletion (GWD) Model

In this section we describe the GWD model that is used to simulate and project changes in groundwater storage in the CV from 1981–2050. This model uses a regional water balance equation as its main building block, augmented with simple empirical relationships between annual precipitation, supply, and demand.

**Figure 6:**
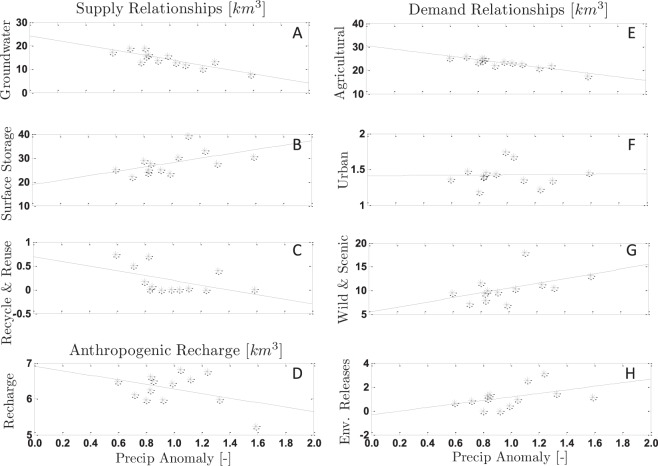
Empirical relationships that describe how each DWR supply and demand variable changes with precipitation. Also shown is the relationship between anthropogenic recharge and precipitation.

Annual supply and demand data from DWR’s California Water Plan was used to constrain our empirical GWD model. The DWR report includes data on the various fresh water supply and demand variables for regions across California. The most recent draft of this plan, the Water Plan Update 2013, provides annual components of water supply from 1998–2010, including surface water (SS), groundwater (GW), and recycled and reused water (RR), as well as annual components of demand, including urban (URB), agricultural (AGR), wild and scenic flows (WS), and environmental managed releases (EnvM). Sub-basin scale data for the Sacramento, San Joaquin and Tulare sub-basins were obtained upon request from the DWR, and the values for each variable were aggregated to represent the CV as a whole. In this paper, demand signifies actual use of water, e.g. agriculture or urban uses, and supply represents the type of water resource, e.g. groundwater or surface water, that was used to meet demand. These different relationships are illustrated by fitted scatter plots in Fig. 6 and they indicate how a given variable responds to different amounts of precipitation in a given year. For example, agricultural demand is greater during dry years or surface water supplies are higher during wet years.

**Figure 7:**
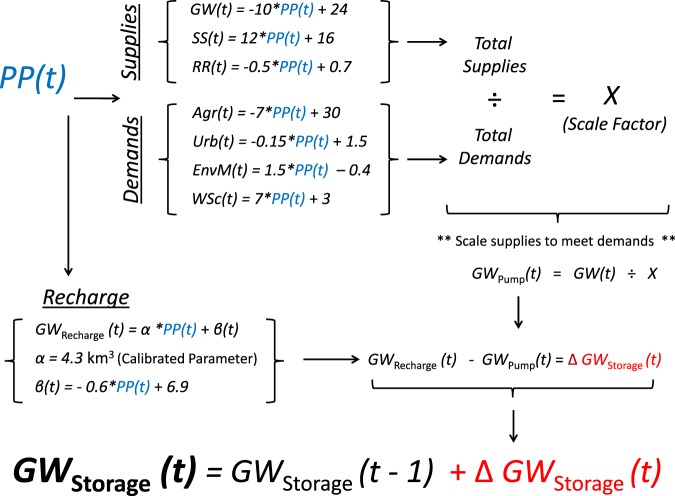
Schematic representing the conceptualized model structure. PP represents the precipitation anomaly each year.

To estimate relative changes in groundwater storage in a given year, the model considers a virtual volume of groundwater, then subtracts the supplied groundwater in that year and adds the volume of recharge calculated for that year. The temporal evolution of the storage in the reservoir is calculated using numerical integration of a simple ordinary differential equation set up with this recipe. The initial storage of the groundwater system, the groundwater storage in 1981, was derived from the USGS CVHM as −36 km^3^. All measurement errors are assumed to be 5 km^3 ^^4^. The GWD model, which uses an annual integration time step, is illustrated schematically with its underlying equations in Fig. 7. We use these empirical relationships to calculate the principal components of a regional water balance for the CV that is then used to determine groundwater storage changes.

Groundwater recharge is difficult to measure in practice, particularly at large spatial scales, due to the complex and highly variable dynamics of the percolation of water from the surface to the subsurface, which can come from a variety of sources, including precipitation, leakage from streams and surface-water bodies, and return flow from irrigated agriculture^13^. Many studies have developed methods for quantifying spatially explicit groundwater recharge^13,32,33^. Yet, these recharge estimates are subject to considerable uncertainty due to limited data on key variables that drive recharge (e.g. soil properties, crop type, irrigation practices). As a result, many existing recharge estimates lack utility for informing model estimates of groundwater storage changes at high spatial and temporal resolutions (Faunt personal communications). In our study, we are interested in modeling annual changes at the basin scale and we use an empirical approach to estimate annual recharge based directly on annual precipitation.

To explicitly estimate recharge in our study we conceptualize two separate sources, one from anthropogenic recharge and the other is directly from precipitation. The anthropogenic recharge term, *β*(*t*), is recharge from return flow from agriculture and percolation from surface water reservoirs. Details on anthropogenic recharge are included from DWR and shown in Fig. 6D. The second term is the recharge from precipitation, and is a process that is parameterized in our model as the variable *α*. This model parameter is calibrated to fit the historic data with the underlying uncertainty, and is estimated to be 4.3 km^3^ per year. This value was estimated using a MCMC method^34^ that searches the feasible parameter space in pursuit of a stationary distribution. This distribution contains the optimum value of the parameter and characterizes its underlying statistical uncertainty. We use a classical Gaussian likelihood function to compare the observed and simulated groundwater storage changes, which tunes the recharge parameter so that the sum of squared residuals of the simulated and observed changes in groundwater storage is minimized.

## Data Availability

Supply and demand information was provided by the California Department of Water Resources (DWR) and is available upon request. The groundwater storage change data (USGS and GRACE) was provided by the UC Center for Hydrologic Modeling, and is also available upon request.
